# Sequence diversity in the A domain of *Staphylococcus aureus *fibronectin-binding protein A

**DOI:** 10.1186/1471-2180-8-74

**Published:** 2008-05-08

**Authors:** Anthony Loughman, Tara Sweeney, Fiona M Keane, Giampiero Pietrocola, Pietro Speziale, Timothy J Foster

**Affiliations:** 1 Department of Microbiology, Moyne Institute of Preventive Medicine, University of Dublin, Trinity College, Dublin, Ireland; 2 Department of Biochemistry, University of Pavia, Pavia, Italy

## Abstract

**Background:**

Fibronectin-binding protein A (FnBPA) mediates adhesion of *Staphylococcus aureus *to fibronectin, fibrinogen and elastin. We previously reported that *S. aureus *strain P1 encodes an FnBPA protein where the fibrinogen/elastin-binding domain (A domain) is substantially divergent in amino acid sequence from the archetypal FnBPA of *S. aureus *NCTC8325, and that these variations created differences in antigenicity. In this study strains from multilocus sequence types (MLST) that spanned the genetic diversity of *S.aureus *were examined to determine the extent of FnBPA A domain variation within the *S. aureus *population and its effect on ligand binding and immuno-crossreactivity.

**Results:**

Seven different isotype forms (I – VII) of the FnBPA A domain were identified which were between 66 to 76% identical in amino acid sequence in any pair-wise alignment. The *fnbA *allelic variants in strains of different multilocus sequence type were identified by DNA hybridization using probes specific for sequences encoding the highly divergent N3 sub-domain of different isotypes. Several isotypes were not restricted to specific clones or clonal complexes but were more widely distributed. It is highly likely that certain *fnbA *genes have been transferred horizontally. Residues lining the putative ligand-binding trench were conserved, which is consistent with the ability of each A domain isotype to bind immobilized fibrinogen and elastin by the dock-latch-lock mechanism. Variant amino acid residues were mapped on a three-dimensional model of the FnBPA A domain and were predicted to be surface-exposed. Polyclonal antibodies raised against the recombinant isotype I A domain bound that protein with a 4 – 7 fold higher apparent affinity compared to the A domains of isotypes II – VII, while some monoclonal antibodies generated against the isotype I A domain showed reduced or no binding to the other isotypes.

**Conclusion:**

The FnBPA A domain occurs in at least 7 different isotypes which differ antigenically and exhibit limited immuno-crossreactivity, yet retain their ligand-binding functions. Antigenic variation of the FnBPA A domain may aid *S. aureus *to evade the host's immune responses. These findings have implications for the development of vaccines or immunotherapeutics that target FnBPA.

## Background

*Staphylococcus aureus *is a commensal of the moist squamous epithelium of the human anterior nares [1]. It is also an opportunistic pathogen that can cause conditions ranging from superficial skin infections to serious invasive diseases such as septicaemia, infective endocarditis and septic arthritis [2].

*S. aureus *can express an array of surface proteins that mediate bacterial binding to plasma proteins and constituents of the extracellular matrix [3], which promote colonization of diverse anatomical sites and contribute to virulence. Many strains can express two distinct fibronectin-binding proteins (FnBPA and FnBPB) which are encoded by the two closely linked genes *fnbA *and *fnbB *[4,5]. However some strains only contain a single gene encoding FnBPA [6]. FnBPA can specifically bind to fibronectin, fibrinogen and elastin [7-9]. FnBPA mediates internalization of *S. aureus *into epithelial and endothelial cells [7,10], promotes rapid aggregation of human platelets [11,12], and is a virulence factor in experimental endocarditis and septic arthritis infection studies [13,14].

Previous work in our group has focused on the binding of the FnBPA N-terminal A domain to fibrinogen and elastin [8,15]. The A domain is predicted to comprise three separately folded sub-domains N1, N2, and N3, similar to the fibrinogen-binding A domains of *S. aureus *ClfA and *Staphylococcus epidermidis *SdrG [16,17]. The X-ray crystal structure of the N23 sub-domains of ClfA and SdrG have been solved in their apo forms and show striking similarities to each other, despite the fact that they are only ~20% identical at the amino acid level [16,17]. Sub-domains N2 and N3 are independently folded in a novel type of immunoglobulin fold (DEv-IgG). The N2 and N3 domains of SdrG are separated by a hydrophobic trench, which binds the fibrinogen β-chain peptide [17]. *In silico *docking studies and mutagenesis revealed that the trench separating N2 and N3 in ClfA is the binding site for the C-terminal fibrinogen γ-chain peptide [16]. A structural model of the FnBPA A domain has a very similar conformation to the solved structures of SdrG and ClfA, including the hydrophobic trench [15]. Residues lining this trench are crucial in binding of the FnBPA to both fibrinogen and elastin [15].

We previously demonstrated that the FnBPA A domain from strain P1 differs substantially from 8325-4 FnBPA, sharing only 73.5% identical amino acids. This was sufficient to create differences in surface-exposed epitopes which affected immuno-crossreactivity, while ligand binding activity was conserved [15]. This study aimed to study a well-characterized strain collection of human origin [18], and human isolates where genomes have been fully sequenced. Five novel FnBPA A domain isotypes were discovered. Many of the isotypes were distributed widely amongst *S.aureus *strains of different MLST genotypes indicating horizontal transfer. Each isotype bound to immobilized fibrinogen and elastin with similar apparent affinities. Polyclonal and monoclonal antibodies raised against the isotype I FnBPA A domain showed reduced binding to other isotypes demonstrating differences in surface exposed epitopes between isotypes.

## Results

### *fnbA *gene variation in *S. aureus *whole-genome sequences

**Figure 1:**
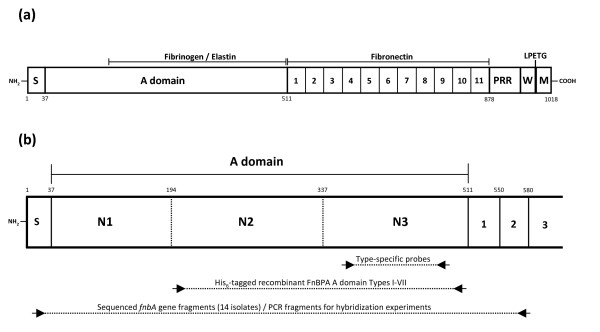
**Schematic representation of FnBPA from *S. aureus *8325-4**. **(a) **The N-terminus of FnBPA contains a signal sequence (S) followed by a 475 amino acid A domain, part of which is the binding site for fibrinogen and elastin. Adjacent to the A domain are eleven fibronectin-binding motifs that span residues 512–878 [27]. The C-terminus of FnBPA contains a proline-rich repeat (PRR), wall (W) and membrane (M)-spanning domains, and the sortase recognition motif LPETG. **(b) **The FnBPA A domain is predicted to comprise three independently-folded sub-domains N1 (37–193), N2 (194–337), and N3 (338–511) [15]. The His-tagged recombinant A domain isotype proteins (I – VII) analyzed during this study comprise the N2 and N3 sub-domains. Type-specific DNA probes used for typing *fnbA *genes from clinical isolates by DNA hybridization contain approximately 300 nucleotides that encode the central portion of the highly divergent N3 sub-domain. The diagram also indicates positions of general primers used for amplifying *fnbA *gene sequences that encode the entire A domain plus flanking sequence. These *fnbA *gene fragments were used as templates in DNA hybridization experiments with type specific probes, and for sequencing from a select number of strains.

We previously reported that a portion of the *fnbA *gene encoding the fibrinogen and elastin-binding A domain (Figure 1) varied substantially in strain P1 compared to the archetypal *fnbA *gene of strain 8325-4 [15], resulting in FnBPA A domains which were 73.5% identical in amino acid sequence. Amino acid substitutions were non-randomly distributed resulting in sub-domain N1 being the most conserved (91.4%), while sub-domains N2 and N3 were more divergent (78.3% and 59.2% identities respectively).

FnBPA A domain sequences from published *S. aureus *genome sequences (COL, USA300, MRSA252, MSSA476, MW2, N315 and Mu50) were analysed to determine if any further variation within the A domain was apparent. Amino acid sequences were aligned in pairwise combinations and the identities calculated. Strains COL, USA300 and 8325-4 contained highly similar FnBPA A domains (one amino acid substitution in COL). This FnBPA A domain is classified as isotype I. These three strains belong to MLST clonal complex 8 (CC8) [19], indicating a close phylogenetic relationship. Strain MRSA252 (ST-36), which belongs to the CC30/39 clonal complex [20], encoded an FnBPA A domain that was divergent from isotype I (75.3% identity) and is here called isotype II. Two CC-1 strains (MSSA476, MW2) contained *fnbA *genes specifying identical A domains, but shared only 76.7% and 80.5% residue identity with isotypes I and II respectively, and are here called isotype III. CC-5 strains (N315, Mu50) specified identical A domains that were very similar (94.5% amino acid identity) to the isotype III A domains of ST-1 strains. Variation between the A domains of ST-1 and ST-5 strains was confined to the N1 sub-domains, while the N23 sub-domains were 100% identical. The classification of FnBPA A domains used in this study (for reasons discussed below) is based on the minimal ligand-binding N23 sub-domains, and therefore the ST-1 and ST-5 FnBPA A domains are classed as isotype III.

The FnBPA A domain from *S. aureus *strain P1 (ST-973), which we analyzed previously [15], was divergent from isotypes I, II and III and is called isotype IV. The divergent N23 sub-domains of isotypes I – IV share between 66.6 and 75.5% identity in any pairwise alignment (Table 3). The N1 sub-domains are more conserved, with average identities of 90.5% between different isotypes. The C-terminal fibronectin-binding motifs 2 – 11 of FnBPA isotypes I – III were highly conserved in the genome sequences indicating that FnBPA A domains alone, in particular the N23 sub-domains, are subject to variation.

### Variation of FnBPA A domains in carriage and clinical *S. aureus *strains

A collection of *S. aureus *strains isolated from carriers and invasive disease patients has previously been analyzed by MLST [18]. Eleven strains representing major clonal complexes (CC15, CC22, CC25, CC45), minor groups (CC12, CC59, CC121), and singletons (ST-55, ST-101) were analyzed. Gene fragments encoding the entire A domain plus some flanking sequence were amplified from genomic DNA by PCR with flanking primers (Figure 1 and Table 2), cloned and sequenced. The deduced A domain amino acid sequences indicated that 10 out of 11 strains encoded N23 sub-domains that are very similar to one of isotypes I – IV described above. Strains from ST-15 and ST-55 encoded isotype I (N23 > 95% identity to 8325-4). Strains from ST-30, ST-59, and ST-101 specfied isotype II (N23 > 97% identity to MRSA252). ST-22, ST-25, and ST-45 strains specified isotype III (N23 > 99% identity to MSSA476). ST-121 and ST-123 strains specified isotype IV (N23 > 96% identity to P1) (Table 1). A novel FnBPA N23 sub-domain sequence was found in *S. aureus *strain 3110 (ST-12) which was between 67.0% and 73.9% identical to A domain isoypes I – IV (Table 3) and is here called isotype V.

**Table 1:** FnBPA A domain (N23) isotype distribution in *S. aureus *strains

*S. aureus *strain	ST	FnBPA isotype	Method of detection
8325-4	8	Type I	*fnbA *gene sequence [5]
USA300	8	Type I	Genome sequence [46]
COL	250	Type I	Genome sequence [47]
382	15	Type I	*fnbA *gene sequence [EMBL: AM749006]
3029	55	Type I	*fnbA *gene sequence [EMBL: AM749009]
964	18	Type I	DNA hybridization
783	15	Type I	DNA hybridization
MRSA252	36	Type II	Genome sequence [20]
3011	30	Type II	*fnbA *gene sequence [EMBL: AM749007]
3111	101	Type II	*fnbA *gene sequence [EMBL: AM749011]
3175	59	Type II	*fnbA *gene sequence [EMBL: AM749013]
114	39	Type II	DNA hybridization
304	39	Type II	DNA hybridization
3132	2	Type II	DNA hybridization
563	37	Type II	DNA hybridization
138	30	Type II	DNA hybridization
316	49	Type II	DNA hybridization
116	9	Type II	DNA hybridization
863	20	Type II	DNA hybridization
3077	17	Type II	DNA hybridization
3084	52	Type II	DNA hybridization
MSSA476	1	Type III	Genome sequence [20]
MW2	1	Type III	Genome sequence [48]
N315	5	Type III	Genome sequence [49]
Mu50	5	Type III	Genome sequence [49]
182	22	Type III	*fnbA *gene sequence [EMBL: AM749015]
205	25	Type III	*fnbA *gene sequence [EMBL: AM749004]
233	45	Type III	*fnbA *gene sequence [EMBL: AM749005]
162	1	Type III	DNA hybridization
617	45	Type III	DNA hybridization
P1	973	Type IV	*fnbA *gene sequence [EMBL: AM749002]
3015	123	Type IV	*fnbA *gene sequence [EMBL: AM749008]
3187	121	Type IV	*fnbA *gene sequence [EMBL: AM749014]
52	188	Type IV	DNA hybridization
3089	97	Type IV	DNA hybridization
3110	12	Type V	*fnbA *gene sequence [EMBL: AM749010]
2	7	Type V	DNA hybridization
402	13	Type V	DNA hybridization
RF122	151	Type VI	Genome sequence (unpublished)
19	10	Type VI	*fnbA *gene sequence [EMBL: AM749003]
3153	207	Type VII	*fnbA *gene sequence [EMBL: AM749012]
H7639	80	Type VII	*fnbA *gene sequence [21]

**Table 2:** Primers

Primer	Sequence (5' – 3')*
Flanking primers	
pfnbA Adom F	CCGAAGCTTGTGAAAAACAATCTTAGGTAC
pfnbA Adom R	CCGGGATCCTATCAATAGCTGATGAATCCG
	
Type-specific probe primers	
pfnbA N3 I F	AAAGGTAGTAATCAAATGG
pfnbA N3 I R	GTTAAGGTATATCCTCTATC
pfnbA N3 II F	GATAGTGTTACTGTAACGG
pfnbA N3 II R	TAGCGATTTTCAGGGTATC
pfnbA N3 III F	TAGTAATTTAGCTGGTGGAC
pfnbA N3 III R	GATATCCCCCATAAACATAG
pfnbA N3 IV F	CAAAGCAAATGGAAATGCTC
pfnbA N3 IVR	AGTTGGTATCCCAAATGAG
pfnbA N3 V F	GAAAGGTAGCAACTCTAATG
pfnbA N3 V R	TGTTAATGTATAGCCGTGG
	
pQE30 expression vector primers	
pfnbApQE II F	CGCGGATCCGGTACAGATGTGACAAGTAAAC
pfnbApQE II R	CCGAAGCTTATATTTTCCATCACCATTAGC
pfnbApQE III F	CGCGGATCCGGTACAGATGTGACAAGTAAAG
pfnbApQE III R	CCGAAGCTTATTTTTACCATTGCCGTCAG
pfnbApQE V F	CCGGGATCCGGCACAGATGTTACAAGTAAAG
pfnbApQE V R	CCGAAGCTTATATTTTCCATCACCATTAGC
pfnbApQE VI F	CTAGGATCCGGCACAGATGTGACAAGTAAAG
pfnbApQE VI R	CCGAAGCTTATTTTTACCATTACCATCAGC
pfnbApQE VII F	CCGGGATCCGGTACAGATGTGACAAGTAAAG
pfnbApQE VII R	CCGAAGCTTATTTTGACCATTACCATCTGC

*restriction endonuclease sites underlined

**Table 3:** Percentage amino acid identities of A domain isotypes I – VII*

	**I**	**II**	**III**	**IV**	**V**	**VI**	**VII**
**I**	100	66.6	69.1	68.4	73.9	68.0	68.2
**II**	66.6	100	73.4	71.8	67.0	65.9	67.2
**III**	69.1	73.4	100	75.5	68.0	69.1	68.5
**IV**	68.4	71.8	75.5	100	72.6	67.2	67.0
**V**	73.9	67.0	68.0	72.6	100	68.6	66.6
**VI**	68.0	65.9	69.1	67.2	68.6	100	68.8
**VII**	68.2	67.2	68.5	67.0	66.6	68.8	100

* Pairwise alignments were performed using the amino acid sequences of the N23 sub-domains of the FnBPA A domain. Sequences used in the alignments were from the following *S. aureus *strains: isotype I – 8325-4; isotype II – MRSA252; isotype III – MSSA476; isotype IV – P1; isotype V – 3110; isotype VI – 19; isotype VII – 3153.

### DNA hybridization analysis using *fnbA *isotype-specific probes

**Figure 2:**
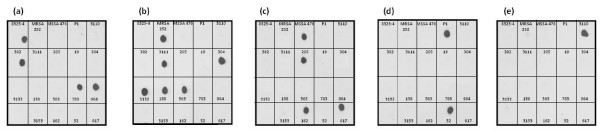
**FnBPA A domain typing of *S. aureus *strains by dot blot hybridization**. Genomic DNA from *S. aureus *isolates was purified, and the *fnbA *gene fragment encoding the entire A domain was amplified by PCR. Five ng purified *fnbA *DNA from each isolate was spotted onto nitrocellulose membranes and probed with DIG-labelled *fnbA *type-specific probes corresponding to isotype I (**a**), isotype II (**b**), isotype III (**c**), isotype IV (**d**) and isotype V (**e**). *fnbA *DNA from isolates 8325-4, MRSA252, MSSA476, P1 and 3110 (top row of blots) were used as controls. The MLST genotypes of strains typed here are given in Table 1.

To determine the distribution of FnBPA A domain isotypes I – V in *S. aureus *strains of different MLST genotypes we used DNA hybridization with isotype-specific probes homologous to a portion of the highly divergent N3 sub-domain. DNA encoding the entire A domain was amplified from strains to be typed (Table 2 and Figure 1). PCR products were spotted onto membranes and hybridized with the DIG-labelled type-specific probes. An example of a hybridization experiment is shown in Figure 2. The probes were shown to be type-specific as each probe only hybridized to the appropriate control *fnbA *fragment (top row of blots). This allowed for rapid typing of multiple strains without the requirement for sequencing. In the example shown, *fnbA *gene fragments of any given strain only hybridized with one probe (Figure 2). The hybridization data for A domain typing of strains is summarized in Table 1.

*fnbA *DNA from *S. aureus *strains 19 (ST-10) and 3153 (ST-207) did not hybridize with any probe (Figure 2), indicating that they express a novel FnBPA isotype. The *fnbA *gene fragments from these two strains were cloned and sequenced, and the deduced A domain protein sequences were compared to the sequences of A domains types I – V. The A domain of *S. aureus *strain 19 (ST-10) was novel and is called isotype VI (N23, 65.9 – 69.1% identical to isotypes I – V; Table 3). It was very similar (97.8% identity) to the FnBPA A domain of bovine strain RF122 (ST-151), the genome sequence of which is available. The A domain of strain 3153 (ST-207) was also novel, and is called isotype VII (N23, 66.6 – 68.8% identical to types I – VI (Table 3)). Isotype VII of strain 3153 is similar (N23, 94.4% identity) to the A domain present in an ST-80 strain [21].

### Distribution of FnBPA isotypes I – VII in strains of different MLST types

**Figure 3:**
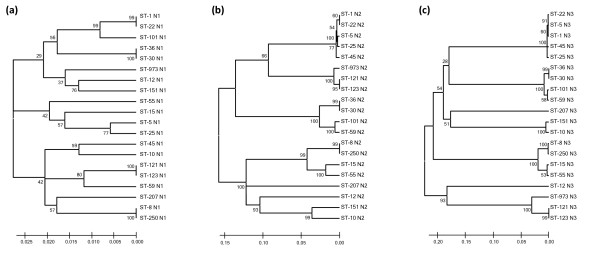
**Evolutionary relationships of *fnbA *N1, N2 and N3 allele sequences**. *fnbA *gene segments encoding the entire FnBPA A domain were sequenced from 14 isolates from divserse MLST genotypes. Strain P1 (ST-973)*fnbA *was sequenced previously [15]. In addition, the *fnbA *gene sequences from MSSA 476 (ST-1); N315 (ST-5); MRSA252 (ST-36); COL (ST-250); RF122 (ST-151) as well as the archetype *fnbA *sequence from 8325-4 (ST-8) were included. The evolutionary history was inferred using the Neighbor-Joining method [43]. Neighbour-joining trees were constructed using *fnbA *DNA sequences encoding the sub-domains N1 (a), N2 (b) and N3 (c) using MEGA 3. The percentage of replicate trees in which the associated taxa clustered together in the bootstrap test (500 replicates) are shown next to the branches [44]. The phylogenetic tree was linearized assuming equal evolutionary rates in all lineages [45]. The evolutionary distances were computed using the Maximum Composite Likelihood method and are in the units of the number of base substitutions per site.

The N1 sub-domain of FnBPA is highly conserved (92.4% amino acid identity between isotypes), while sub-domains N2 and N3 are more divergent (77.5% and 66.2% average amino acid identity, respectively). Neighbour joining (NJ) trees based on DNA sequences of the *fnbA *allelic variants demonstrate N2 and N3 sequences appear to have co-evolved. For example, strains of ST-36, ST-30, ST-59 and ST-101 have *fnbA *genes encoding isotype II A domains that are greater than 97% identical. In the *fnbA *N2 NJ tree (Figure 3b) and the *fnbA *N3 NJ tree (Figure 3c) sequences from these strains clustered together. Similarly, strains carrying isotype I (ST-8, ST-250, ST-15, ST-55), isotype III (ST-1, ST-5, ST-22, ST-25, ST-45), isotype type IV (ST-121, ST-123, ST-973), or isotype VI (ST-10, ST-151) clustered similarly according to N2 and N3 *fnbA *sequence (Figures 3b and 3c). Therefore N2 and N3 sub-domains are always associated in a single combination, which is likely to conserve ligand binding functions. For this reason, we have defined isotypes based on the highly variable N23 sub-domain amino acid sequences.

The N1 sub-domain sequences appear to have evolved in distinct manner from the N23 sub-domain sequences (Figure 3). For example, isotype III (N23) A domains of MSSA476 (ST-1) and N315 (ST-5) are 100% identical, while their N1 sub-domains have only 86.2% identity. The DNA sequences encoding N1 sub-domains are generally highly conserved, reflected by the low number of substitutions per site (Figure 3a), and their phylogeny does not appear to match that of the N2 or N3 sub-domain encoding sequences (Figure 3), indicating a different pattern of evolution not related to ligand binding activity. It should be noted that in strains of the same ST (e.g. ST-1 strains MSSA476 and MW2), or strains within the same clonal complex (e.g ST-250 (COL) and ST-8 (8325-4) from CC8) the entire A domains (N123) are very highly conserved.

**Figure 4:**
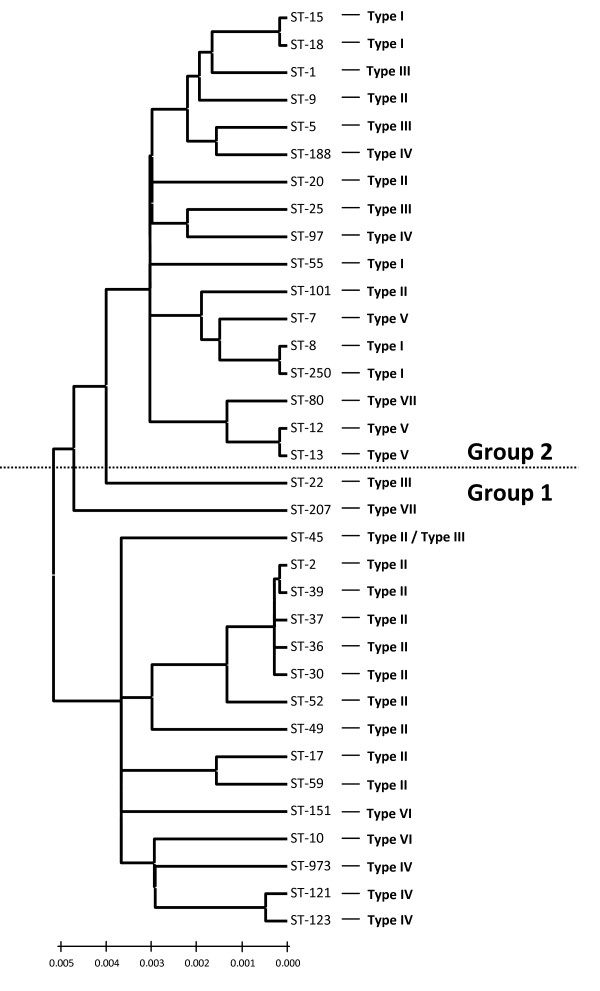
**Neighbour-joining phylogentic tree of concatenated MLST allele sequences**. Concatenated MLST allele sequences representing each strain were downloaded from the MLST database [39] and used to generate a neighbour joining tree using MEGA 3 [40]. The A domain isotypes carried by strains of each MLST genotype, determined by sequencing and hybridization analysis, are indicated. FnBPA A domain types from strains of ST-80 and ST-45 were determined from sequence data from a previous study of polymorphic genes in *S. aureus *[21]. The dashed line indicates the separation of the MLST genotypes into Groups 1 and 2, which is based on sequence data from MLST alleles and other unlinked loci [23].

Figure 4 shows a NJ phylogenetic tree based upon the concatenated sequences of the 7 alleles used for MLST analysis. As MLST reflects the evolution of the stable core genome [22], this tree depicts the phylogenetic relatedness of the *S. aureus *strains studied here (Table 1). It is separated into two major groupings which reflect previous phylogenetic analysis using MLST alleles plus additional sequences from unlinked loci [23]. Some *fnbA *allelic variants were not restricted to specific clones or lineages, but were widely distributed throughout the population. For example, several strains from CC30/39 (ST-30, ST-36, ST-37, ST-39, ST-2) contained FnBPA isotype II (Figure 4), as did distantly related strains from Group 2 (ST-9, ST-20, ST-101). Also isotype III was found in Group 2 strains of ST-1, ST-5 and ST-25 and in Group 1 strains of ST-22 and ST-45 (Figure 4). Conversely, closely related types such as ST-80 and ST-12 in Group 2 have *fnbA *genes encoding isotypes VII and V respectively. The *fnbA *allele of the ST-80 strain H7639 was determined from a recent study of polymorphic genes in *S. aureus *[21]. These data suggest that *fnbA *genes have occasionally been horizontally transmitted between strains of different lineages.

### Location of variant amino acid residues on molecular models of the FnBPA A domain

The FnBPA A domain of *S. aureus *8325-4 has a similar organization to the fibrinogen-binding A domain of *S. aureus *ClfA, the X-ray crystal structure of which has been solved [16]. A 3D model of the isotype I A domain of FnBPA closely resembles the ClfA structure [15]. Variant residues between isotypes I and IV were predicted to be mostly exposed on the surface of the protein model [15]. A similar analysis was performed here which showed that the variant residues of isotypes II, III, V, VI and VII were predicted to be exposed on the A domain surface (data not shown). Residues that were identified by mutagenesis as important in fibrinogen and elastin binding by the Type I A domain [15] were highly conserved across all isotypes. Structural models of isotypes II – VII were generated using the web based PHYRE program (data not shown) based on the crystal structure of ClfA. Each showed striking similarities to the isotype I model [15] (data not shown).

### Binding of FnBPA A domains isotypes I – VII to immobilized fibrinogen and elastin

**Figure 5:**
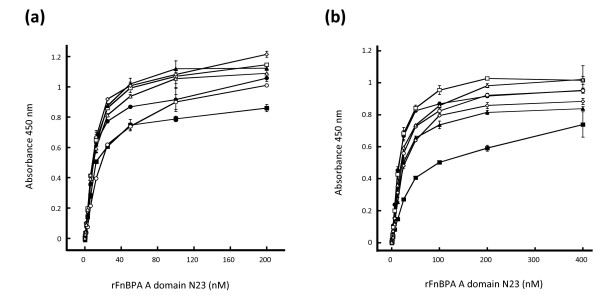
**Binding of recombinant A domain isoypes I – VII to immobilized fibrinogen and elastin**. Microtitre dishes were coated with human fibrinogen (**a**) or human aortic elastin (**b**). Wells were blocked and then incubated with recombinant A domain proteins isotype I (closed squares), isotype II (closed circles), isotype III (open squares), isoype IV (open circles), isoype V (closed triangles), isoype VI (open diamonds), and isoype VII (open triangles) at the indicated concentrations. Bound A domain proteins were detected by incubation with mouse anti-hexahistidine monoclonal antibody 7E8, then HRP-conjugated goat anti-mouse IgG antibodies followed by TMB substrate. Graphs are representative of three separate experiments.

The minimum ligand binding N23 sub-domains of isotype I and isotype IV were studied previously [15]. Here the N23 sub-domains of isotypes II, III, V, VI and VII were also expressed. Each isotype bound to immobilized fibrinogen and elastin in a dose-dependent and saturable manner (Figure 5). The apparent affinity of each isotype for fibrinogen was very similar with a half maximum binding concentration of ca. 10 nM (Figure 5a). The isotype I A domain bound elastin with a half maximum of 45 nM whereas isotypes II – VII had a higher affinity with a half maximum at 15 – 20 nM (Figure 5b). This is consistent with the previous report for isotype I and IV [15]. The conservation of binding activity across all isotypes indicates that ligand binding by the FnBPA A domain is biologically important for *S. aureus*.

### Binding of antibodies to isotypes I – VII

**Figure 6:**
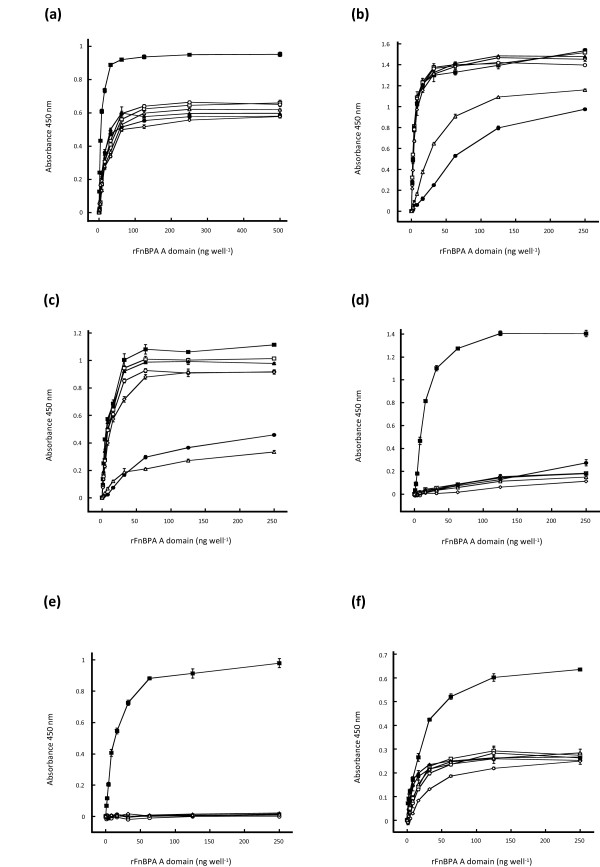
**Binding of polyclonal and monoclonal anti-isotype I antibodies to A domain isotypes I – VII**. Microtitre dishes were coated with A domains isoype I (closed squares), isotype II (closed circles), isotype III (open squares), isotype IV (open circles), isotype V (closed triangles), isotype VI (open diamonds), and isotype VII (open triangles) at the indicated concentrations. Wells were then blocked and then incubated with (**a**) polyclonal rabbit anti-isotype I A domain antibodies, or mouse monoclonal anti-isotype I A domain antibodies 1F9 (**b**), 1G8 (**c**), 7C5 (**d**), 1E6 (**e**), and 7B7 (**f**). Bound antibodies were detected with either HRP-conjugated goat anti-rabbit IgG antibodies (**a**) or HRP-conjugated goat anti-mouse IgG antibodies (**b-f**), followed by TMB substrate. Graphs are representative of three separate experiments.

The majority of variant residues between different A domain types were predicted to be exposed on the surface. This might create differences in surface-exposed epitopes that affect antigenicity. We previously demonstrated that polyclonal antibodies raised against isotype I reacted less efficiently with isotype IV by Western immunoblotting [15]. Here the ability of polyclonal anti-isotype I antibodies to bind different isotypes was measured by ELISA. Each protein coated the microtitre wells with equal efficiency when detected with anti-hexahistidine monoclonal antibody 7E8 (data not shown). Rabbit anti-isotype I antibodies had a 4 – 7 fold lower affinity for isotypes II – VII compared to isotype I (Figure 6a). Thus amino acid variation creates differences in surface-exposed epitopes on the A domain molecule that affect immuno-crossreactivity.

Several mouse monoclonal antibodies (1F9, 1G8, 7C5, 1E6 and 7B7) directed against isotype I were tested by ELISA for binding to each isotype. Antibodies 7C5 and 1E6 bound efficiently to isotype I but showed little binding to isotypes II – VII (Figure 6d and 6e) indicating that the 7C5 and 1E6 epitope is only present on isotype I. Similarly, monoclonal antibody 7B7 reacted strongly with isotype I, but bound poorly to isotypes II – VII (Figure 6f) indicating that only part of the 7B7 epitope is present on A domains II – VII. 1F9 and 1G8 had a high affinty for isotypes I, III, IV, V and VI and a lower affinity for isotype II and VII (Figure 6b and 6c) indicating that they recognise a relatively conserved epitope which is partially absent in isotypes II and VII. It is possible that the IF9 and 1G8 bind the same or overlapping epitopes.

We previously reported that monclonal antibodies 7C5, 1E6, 1F9 and 1G8 failed to bind to isotype IV by Western blotting and that they bound weakly to isotype I [15]. 1E6 and 7C5 did not react with the isotype IV protein by ELISA while 1F9 and 1G8 reacted well (Figure 6). This discrepancy may be explained by inefficient refolding after denaturation in Western immunoblotting.

## Discussion

We have reported on the substantial sequence diversity in *fnbA *genes amongst *S. aureus *isolates giving rise to 7 isoforms of the FnBPA A domain, which are between 24.5 and 34.1% divergent in amino acid sequence. There was strong conservation of residues known to be involved in ligand binding by isotype I, which were predicted to line the putative ligand-binding trench in a 3D structural model [15]. This conservation is consistent with the ability of each isotype to bind immobilized fibrinogen and elastin with similar affinities. Ligand-binding by the FnBPA A domain is therefore likely to be biologically important to *S. aureus *in vivo.

The variant residues between isotypes were mapped on the 3D-structural model of the A domain, and the vast majority were predicted to be surface exposed. Antibody binding experiments were performed to demonstrate that variation between isotypes affected their antigenicity. Polyclonal antibodies against isotype I had reduced affinity for isotypes II – VII. Some monoclonal antibodies raised against isotype I had little or no affinity for all other isotypes, while others reacted well with some isotypes and not others. Sequence variation has therefore created differences in epitopes between different FnBPA A domain isotypes.

FnBPA has been documented as an important virulence factor of *S. aureus*, contributing to experimental endocarditis and septic arthritis infections [13,14]. Carriage of the *fnbA *gene by *S. aureus *was found to be significantly more common in invasive isolates compared to carriage isolates [24], suggesting a role in pathogenicity. Studies on the immune responses of individuals exposed to invasive *S. aureus *infections have shown that serum antibody titres against FnBPA rise as a result of infection [25,26]. Antibodies directed against the A domain were greatly elevated in sera of patients who had endocarditis caused by *S. aureus*, compared to sera of healthy controls [25]. This indicates that FnBPA is expressed in vivo, is immunogenic, and that the host mounts a robust immune response against this antigen. The evolution of the FnBPA A domain into distinct isotypes with different antigenicities may have been driven by the need to evade the host immune responses during infection.

It is interesting to note that variation amongst FnBPA appears to be confined to the A domain. Analysis of entire *fnbA *genes from sequenced genomes reveals that the FnBPA C-terminal fibronectin-binding motifs 2 – 11 are highly conserved in amino acid sequence. This is an unfolded domain lacking secondary structure, and undergoes a conformational change to an ordered β-sheet structure upon binding to fibronectin [27]. Anti-FnBPA antibodies are generated during infection that recognize both the A domain and the fibronectin-binding motifs [25,28]. The antibodies directed against the fibronectin-binding motifis predominantly recognize epitopes formed in the β-sheet structure when fibronectin is bound to FnBPA. Ligand-induced binding site (LIBS) antibodies are not function-blocking [28]. Indeed, it has been suggested that LIBS antibodies may be beneficial by stabilizing fibronectin binding and thus enhancing bacterial adherence to the host tissue [29]. In contrast the FnBPA A domain is likely to be projected away from the bacterial cell and to be recognized by opsonizing antiodies. However, there is no obvious explanation as to why FnPBA A domains have diverged so much whereas proteins with similar A domains such as ClfA and ClfB are much less divergent [21].

Individuals who carry *S. aureus *have an increased chance of developing invasive disease that is usually caused by the carriage strain, but have a lower mortality rate than non-carriers that develop *S. aureus *bacteremia [30,31]. Carriers have been shown to mount an immune response against the superantigens that are specifically expressed by their colonizing strain [32]. Generation of a strain-specific immune response might explain the improved prognosis of carriers compared to non-carriers [32]. A similar phenomenon might occur with FnBPA, whereby carriers mount a specific immune response to the FnBPA isotype expressed by their colonizing strain and prime the immune system to mount a robust anti-FnBPA antibody response during infection. Conversely, FnBPA variation may help strains to infect individuals previously exposed to a different isotype.

The *fnbA *allelic variants encoding many of the 7 A domain isotypes were widely distributed. Several isotypes were not restricted to specific clones or clonal complexes. Indeed, the same A domain isotype was often found in strains that are distantly related based upon the phylogenetic analysis. This suggests that horizontal transfer of *fnbA *loci has occurred. Similarly, there is evidence of horizontal transfer of staphylocoagulase serotypes [33]. For example, the *fnbA *allelic variants found in strains representative of CC1, CC5, CC22, CC25 and CC45 encoded isotype III A domains that were extremely similar (> 99% amino acid identity), yet these strains belong to distantly related clonal complexes. Indeed, the *fnbA *allele we sequenced from a ST-22 strain was identical to the *fnbA *gene of ST-1 strains MSSA476 and MW2, which suggests a recent recombination event. Further evidence for horizontal transfer is the presence of two different FnBPA A domains isotypes in strains of the same MLST genotye (ST-45). We found allelic variants encoding isotype III in ST-45. The study of Kuhn *et al *[21] describes two ST-45 strains carrying *fnbA *alleles 11 and 12, which encode isotype II A domains, while other ST-45 strains were found carrying alleles 14 and 16, which encode type III A domains. This shows that *fnbA *genes encoding isotype II or III A domains are present in ST-45 strains. It has been suggested that CC45 and CC30/39 share a common ancestor [23], so perhaps ancestral ST-45 strains specified isotype II as in CC30/39 and that horizontal transfer was responsible for establishing the isotype III A domain in certain ST-45 strains.

The FnBPB A domain appears to have evolved in the same way as FnBPA described here. Analysis of the FnBPB A domains obtained from genome sequences has identified three FnBPB isotypes and a further four FnBPB A domain isotypes were identified by hybridization and DNA sequencing (F.M. Burke and T.J.F., unpublished data). Evidence for interstrain recombination came from the occurrence of different FnBPA and FnBPB isotype combinations in different strains.

Amongst the FnBPA A domain, it appears that the minimum ligand-binding truncate (sub-domains N2 and N3) have evolved in parallel into at least 7 distinct isotype groupings. This is clearly illustrated in the neighbour-joining phylogenetic trees based upon *fnbA *sub-domain sequences, where a particular N2 sub-domain is always found in association with a particular N3 sub-domain. The phylogeny of the N1 sub-domain appeared to differ from that of the N23 sub-domain. For example, the amino acid sequence of the FnBPA N23 sub-domain from N315 and Mu50 (ST-5; isotype III) is identical to the N23 sub-domain from MSSA476 and MW2 (both ST-1). However, their N1 sub-domains are only 86.2% identical. Perhaps recombination may have occurred within *fnbA *genes to switch segments encoding domains N23 and N1.

## Conclusion

We have identified seven isotypes of the N-terminal A domain of FnBPA in a collection of *S. aureus *strains representing the genetic diversity of the species. Isotypes are on average 29.3% divergent in amino acid sequnce. Each isotype is functional with respect to ligand-binding activity, but they differ in immuno-crossreactivity, which may play a role in immune evasion. Isotypes are distributed randomly throughout the *S. aureus *population, and homologous recombination may be involved in dissemination of *fnbA *loci amongst *S. aureus*. These data have implications for the FnBPA A domain as a target for a vaccine or immunotherapeutic.

## Methods

### Bacterial strains

*Escherichia coli *strains were cultivated on L-agar and L-broth with shaking at 37°C. *E. coli *strain XL1-Blue (Stratagene) was routinely used for cloning. *E. coli *Topp 3 (Qiagen) was used for the expression of recombinant FnBPA A domain proteins. Ampicillin (100 μg ml^-1^) was incorporated into growth media where appropriate. *S. aureus *strains (Table 1) were isolated from individuals from Oxfordshire, U.K, and had previously been characterized by MLST [18]. *S. aureus *strains were cultivated on TSA agar and BHI broth.

### Preparation of genomic DNA from *S. aureus*

Cultures were grown in 5 ml BHI broth at 37°C with shaking for 24 h. Genomic DNA was prepared using the Edge Biosystems Bacterial Genomic DNA purification kit with the addition of 100 μg ml^-1 ^lysostaphin at the cell lysis step. DNA pellets were washed with 70% v/v ethanol, air-dried, and resuspended in 100 μl of TE buffer (10 mM Tris-HCl, pH 8.0; 1 mM EDTA).

### Cloning of *fnbA *gene fragments

Primers pfnbA Adom F1 and pfnbA Adom R1, corresponding to DNA encoding the signal sequence and fibronectin binding domain 2 respectively (Table 2 and Figure 1), were designed from conserved sequences in *fnbA *genes from publicly available *S. aureus *genomes. DNA encoding the entire FnBPA A domain along with flanking sequences was amplified, cleaved with *Bam*HI and *Hind*III (Boehringer Mannheim) at restriction sites incorporated into the primers, ligated using T4 DNA ligase (Roche) to pBluescript KS+ DNA cleaved with the same enzymes, and finally transformed into *E. coli *XL1-Blue cells using standard procedures [34]. Transformants harbouring cloned *fnbA *gene fragments were selected on L-agar with ampicillin and 80 μg ml^-1 ^of the chromogenic substrate 5-bromo-4-chloro-3-indolyl-β-D-galactoside (Melford Laboratories, Ipswich, U.K.). Plasmid DNA was purified from transformants using the Favorgen Plasmid DNA Extraction Mini Kit (Favorgen Biotech Corp). The cloned *fnbA *gene fragments were sequenced using T3 and T7 primers by GATC Biotech AG (Germany). The EMBL accession numbers for the sequences reported in this paper are AM749002 – AM749015.

### DNA hybridization using *fnbA *type-specific probes

Genomic DNA samples from clinical isolates were used as the template in PCR reactions with primers pfnbA Adom F1 and pfnbA Adom R1 (Table 2 and Figure 1) to amplify the entire coding region of the FnBPA A domain. PCR amplimers were purified using the Wizard SV Gel and PCR Clean-Up System kit (Promega) Five ng of *fnbA *DNA was spotted onto positively-charged nylon membranes (Roche) and allowed to air-dry. Membranes were incubated for 5 min on blotting paper soaked in denaturation solution (1.5 M NaCl, 0.5 M NaOH), 5 min in neutralization solution (1.5 M NaCl, 1 M Tris-HCl, pH 7.4), and finally for 15 min on blotting paper soaked with 2× SSC solution (300 mM NaCl, 30 mM tri-sodium citrate). DNA was fixed on the membranes by incubation at 120°C for 30 min. Membranes were incubated for 2 h at 68°C in pre-hybridization solution (5× SSC, 0.1% w/v N-lauroylsarcosine, 0.02% w/v SDS and 1× Blocking reagent (Roche)). DIG-labelled probes complementary to DNA encoding isotype I – V N3 sub-domains were synthesised by PCR using type-specific probe primers (Table 2 and Figure 1) and PCR DIG labelling mix (Roche). Probes were denatured by boiling at 95°C for 10 min, diluted in pre-hybridization solution and incubated with nylon membranes for 18 h at 68°C. Following hybridization, the membranes were washed twice with 2× SSC/0.1% w/v SDS at room temperature followed by two washes with 0.5× SSC/0.1% w/v SDS at 68°C for 20 min. Membranes were equilibrated for 30 min in maleic acid buffer (100 mM maleic acid, 150 mM NaCl, pH 7.5), and bound DIG-labelled probes were detected by incubation for 30 min with alkaline phosphatase-conjugated anti-DIG antibody (Roche) diluted 1:10,000 in maleic acid buffer. After washing twice with maleic acid buffer containing 0.3% v/v Tween 20, the chemiluminescence substrate CSPD (Roche) was used to detect bound anti-DIG antibodies and membranes were exposed to X-OMAT UV Plus Film (Kodak).

### Expression of recombinant FnBPA A domain proteins

pQE30 expressing the minimal ligand-binding FnBPA A domain truncate (N23: residues 194 – 511) from *S. aureus *8325-4 and *S. aureus *P1 (residues 194 – 512) were described previously [15]. pQE30 expressing His-tagged N23 A domains of isotypes II, III, V, VI and VII were constructed with pQE30 vector primer pairs (Table 2 and Figure 1) and genomic DNA templates from strains MRSA252 (ST-36), MSSA476 (ST-1), 3110 (ST-12), 19 (ST-10) and 3153 (ST-207), respectively. Each construct was verified by sequencing (GATC Biotech AG, Germany) and transformed into *E. coli *Topp 3 for protein expression and purification. Proteins were purified by Ni^2+ ^chelate chromatography [34]. Concentrations were determined using the BCA Protein Assay Kit (Pierce). Proteins were dialysed against PBS for 24 h at 4°C, aliquoted and stored at -70°C.

### Binding of recombinant FnBPA proteins to immobilized elastin and fibrinogen

Human aortic elastin (Elastin Products Company) and human fibrinogen (Calbiochem) were coated onto Nunc 96-well microtitre dishes (1 μg protein well^-1^) for 18 h under UV light and at 4°C respectively. Wells were washed three times with phosphate-buffered saline (PBS) containing 0.05% v/v Tween 20 (PBST) and blocked with 2% w/v bovine serum albumin (BSA; Sigma) in PBST buffer (BSA-PBST) for 2 h at 37°C. Following three washes with PBST, various concentrations of purified FnBPA isotype proteins in 2% w/v BSA-PBST were added to the wells and incubated at room temperature for 1 h with shaking. Wells were washed three times with PBST buffer and bound proteins were detected with monoclonal antibody 7E8 (90 ng well^-1^) that recognises the N-terminal hexahistidine fusion tag. Following incubation for 1 h with shaking at room temperature, the wells were washed three times with PBST followed by 100 μl per well of goat-anti-mouse antibodies conjugated to horseradish-peroxidase (HRP, Dako; Denmark) diluted 1:2000 in 2% w/v BSA-PBST. After incubation for 1 h at room temperature, wells were washed three times with PBST, and bound HRP-conjugated antibodies were detected with 10 μg per well of 3,3',5,5'-tetramethylbenzidine (TMB; Sigma) in 0.05 M phosphate-citrate buffer containing 0.006% (v/v) hydrogen peroxide. After incubation at room temperature for 5 min the reaction was stopped by adding 50 μl of 2 M H_2_SO_4_. The absorbance at 450 nm was measured with an ELISA plate reader (Multiskan EX, Labsystems).

### ELISA assays

Various concentrations of recombinant FnBPA A domain proteins in PBS were coated onto Nunc 96-well microtitre dishes for 18 h at 4°C. Wells were washed and blocked with BSA for 2 h as described above. Following three washes with PBST, 100 μl of anti-FnBPA A domain antibodies diluted in BSA-PBST (1.8 μg polyclonal IgG ml^-1^; 2.5 μg monoclonal IgG ml^-1^), were added to each well and incubated for 1 h at room temperature with shaking. Polyclonal and monoclonal antibodies 7C5, 1E6, 1F9 and 1G8 raised against the isotype I A domain of FnBPA from *S. aureus *8325-4 were described previously [8,11,15]. Monoclonal antibody 7B7 was generated by immunizing mice with recombinant Type I FnBPA A domain (N123 sub-domains) as described previously [11]. After 1 h incubation the wells were washed three times with PBST. Goat anti-rabbit IgG-HRP conjugated antibodies or goat anti-mouse IgG-HRP conjugated antibodies (Dako, Denmark), each diluted 1:2000 in BSA-PBST, were added to the wells and incubated for 1 h. After washing three times with PBST, bound HRP-conjugated antibodies were detected as described above.

### Bioinformatic and phylogenetic analysis of FnBPA A domain isotypes

Protein sequences were aligned in pairwise combinations to calculate amino acid identity using the ExPASY SIM alignment tool [35]. Alignments of multiple protein sequences to view areas of conservation amongst A domains were performed using T-Coffee [36]. Theoretical 3D models of A domain isotpyes I – VII were generated using PHYRE Protein Fold Recognition Server [37]. Protein structure files were viewed using PyMOL [38], which allowed visualization of the distribution of variant residues between isotypes. The concatenated MLST allele sequences of *S. aureus *strains were downloaded from the MLST database [39]. Phylogenetic and molecular evolutionary analyses of *fnbA *gene sequences and concatenated MLST allele sequences were conducted using MEGA version 3.1 [40]. The overall relatedness of the MLST population of *S. aureus *was visualized using eBURST [41,42].

## Authors' contributions

AL carried out cloning of *fnbA *genes for sequencing and protein expression, DNA and amino acid sequence analysis, purification of recombinant A domain proteins, ELISA experiments, and drafted the manuscript. TS carried out DNA hybridization experiments. FMK carried out cloning of *fnbA *genes for recombinant expression and provided polyclonal antisera to the isotype I A domain. GP and PS were responsible for production of monoclonal antibodies against the isotype I A domain. TJF conceived and coordinated the study, and helped to draft the manuscript. All authors read and approved the final manuscript.
